# The international spill over effect of American economy on China’s macro-economy based on MCMC-Gibbs sampling algorithm

**DOI:** 10.1371/journal.pone.0293909

**Published:** 2023-11-03

**Authors:** Jiamu Hu

**Affiliations:** D’Amore-McKim School of Business, Northeastern University, Boston, Massachusetts, United States of America; University of Almeria, SPAIN

## Abstract

China’s export benefits from the significant fiscal stimulus in the United States. This paper analyzes the global spillover effect of the American economy on China’s macro-economy using the Markov Chain Monte Carlo (MCMC)-Gibbs sampling approach, with the goal of improving the ability of China’s financial system to protect against foreign threats. This paper examines the theories of the consequences of uncertainty on macroeconomics first. Then, using medium-sized economic and financial data, the uncertainty index of the American and Chinese economies is built. In order to complete the test and analysis of the dynamic relationship between American economic uncertainty and China’s macro-economy, a Time Varying Parameter-Stochastic Volatility-Vector Autoregression (TVP- VAR) model with random volatility is constructed. The model is estimated using the Gibbs sampling method based on MCMC. For the empirical analysis, samples of China’s and the United States’ economic data from January 2001 to January 2022 were taken from the WIND database and the FRED database, respectively. The data reveal that there are typically fewer than 5 erroneous components in the most estimated parameters of the MCMC model, which suggests that the model’s sampling results are good. China’s pricing level reacted to the consequences of the unpredictability of the American economy by steadily declining, reaching its lowest point during the financial crisis in 2009, and then gradually diminishing. After 2012, the greatest probability density range of 68% is extremely wide and contains 0, indicating that the impact of economic uncertainty in the United States on China’s pricing level is no longer significant. China should therefore focus on creating a community of destiny by working with nations that have economic cooperation to lower systemic financial risks and guarantee the stability of the capital market.

## 1. Introduction

Between 1978 and 2010, China’s GDP increased from 1.7% to 9.5% of the world economy at market exchange rates. This is because the real income of most developing countries is overestimated as a result of the price difference between traded and non-traded commodities. Over the past two decades, China has developed into the second-largest economy in the world and a prominent role in the global economy. The rapid growth of China’s GDP has altered the distribution of economic activities around the world [1–3]. Although China’s trade share has decreased since 2022, the export of foreign demand has held steady. According to the most recent data from the World Trade Organization, China had a global market share of 14.09% for commodity exports in the first half of 2022, down 0.47 percentage points from the previous year. This decline is primarily attributable to the restart of production in other nations and the crowding-out effect of skyrocketing energy prices. The price of consumer products has decreased globally as a result of China’s exports, which have made it the main hub of trade among industries [4, 5].

Due to the world’s rising financial and economic interconnectedness, China and the United States’ trade and financial ties are strengthening. The stock market crash, the downfall of financial institutions, and an economic slowdown were only a few of the serious effects of the US subprime mortgage financial crisis. The subprime mortgage crisis in the United States, which began in the real estate sector and expanded to other economic sectors, had an impact on the global financial system [6–8]. Financial institutions have suffered large losses in many different countries, particularly in Europe. Because investors in these institutions are often risk averse, there is a large capital outflow from emerging market countries that have minimal direct engagement with the real estate sector. The impact of this crisis on the economic outlook and risk-taking behavior around the world has also affected China. The slow rate of economic recovery in developed countries restricts China’s export growth. The country’s economy will eventually continue to grow sustainably as a result of China’s market expansion [9]. In terms of interest rate channels, as China’s economic volume continues to converge with that of the United States, or as the GDP ratio between China and the United States is rising, it will adversely regulate the reverberation effect of interest rate changes in the United States on China’s interest rate. In other words, China has more autonomy in determining its own interest rate policy in the face of the effect of interest rate changes in the United States [10–12].

The United States and China’s futures trading dwarfs the world’s current foreign exchange market. This illustrates that China and the United States have very strong financial ties, ties that will further deepen as a result of the fake economy and the extraordinary increase in cash movement between the two countries. The internal and external settings in which China’s exports and cross-border capital flows are placed continue to be disturbing despite the fact that the country’s overall exports are increasing.

The international spillover effect of the United States on China is mainly based on the following reasons. First, under the background of globalization, the international economic ties are getting closer and closer, and the interaction between economies has become more significant. As the two largest economies in the world, the economic relationship between the United States and China is of great significance. Second, as one of the largest economies in the world, the economic performance of the United States has a wide and far-reaching impact on the global economic structure. Especially in the case of the implementation of major fiscal stimulus policies in the United States, this impact is even more significant. The research of Giovanni et al. (2022) provided an important insight into this issue [13]. Their research aimed to analyze the spillover effects of the US economy on the global economy, including the impact on China. By using econometric models and empirical data, they demonstrated the transmission mechanism and effect of American economic policies and fluctuations on China’s macro-economy. The results showed that the uncertainty and fluctuation of American economy have a significant impact on China’s price level and macroeconomic activities. Based on the research of Giovanni et al., it can be understood that exploring the international spillover effect of the United States on China is of great significance for understanding global economic ties, risk transmission and macroeconomic policy formulation. A thorough study of the impact of U.S. economic fluctuations on China’s economy can help China strengthen its financial system’s ability to resist external risks and formulate more effective macroeconomic policies to stabilize economic growth and meet the challenges from the U.S. economy. Therefore, it is necessary to explore the international spillover effect of the United States on China, and it is of great guiding significance to the macroeconomic development and stability of China. This paper’s investigation of how the American economy affects China’s macroeconomics serves two major objectives. First, improve quantitative study on the effects of cross-border economic spillover and ascertain the influence of the American economy on China’s domestic macro-economy. Second, identify the channels by which the American economy crosses international borders and offer a theoretical framework on which government organizations can base their decisions about how to strengthen the resilience of China’s financial and economic system to external threats.

## 2. Materials and methods

### 2.1 Macroeconomic effects of economic uncertainty

Currency functions as a transactional instrument as well to deal with future uncertainty. People can retain more cash and reduce risks by increasing the liquidity of their assets. The investments made by business owners are also impacted by the prospect of future instability. When the expectation of uncertainty results in a decline in the marginal efficiency of capital, business owners will reduce their investment. Because there are numerous ways that people think about uncertainty, there are various explanations for doubt. Economic uncertainty largely has two effects on issues at the macro level. On the one hand, it investigates how economic unpredictability impacts the systemic financial risk spread mechanism, while on the other hand, it investigates how unpredictability in the economy influences and forecasts macroeconomics. Economic instability will have an adverse effect on the macroeconomy. Following the negative consequences of the financial crisis, the slow macroeconomic recovery is largely due to the unpredictability of economic policies in various countries. The strength of the macroeconomy and economic uncertainty are negatively connected, and the effectiveness of relevant policies is also negatively correlated with economic uncertainty [14, 15]. The uncertainty of economic policy, which is not what is creating China’s economic volatility, has restrained price growth.

The economic topic is the research subject for macroeconomic uncertainty, but there is uncertainty because people only have partial knowledge and cannot accurately predict the outcomes of the future [16]. That is, an unpredictable component of each economic indicator is the difference between its real value and its predicted value. While macroeconomic instability is a complex concept. Only a small percentage of the overall uncertainty is captured by the unexpected component of a single economic indicator, however the combination of unpredictable and changing components in multivariate economic indicators can better represent macroeconomic uncertainty. Production, investment, and consumption must all be considered in the evaluation. According to research on the relationship between economic uncertainty and monetary policy, economic uncertainty undermines expansionary policy, whereas expansionary fiscal policy, in turn, lowers the degree of economic uncertainty. Combining the two reveals that fiscal policy is unable to mitigate the adverse impacts of economic volatility, which are more evident.

Due to the uncertainty impact of risk aversion, consumers may increase their precautionary savings, which would reduce consumption expenditure. This effect might promote economic growth in the short term, but its long-term implications are unknown [17–19]. Theoretically, this is because there is a chance that decreasing consumption and increasing savings may lead to greater investment, which will aid long-term growth. In most open economies, a portion of the extra savings will go abroad, reducing domestic demand. In smaller and more open economies, the increase in uncertainty brought on by the national currency’s devaluation will therefore slow economic growth.

### 2.2 Cross-border spillover effect of economic uncertainty

**Fig 1:**
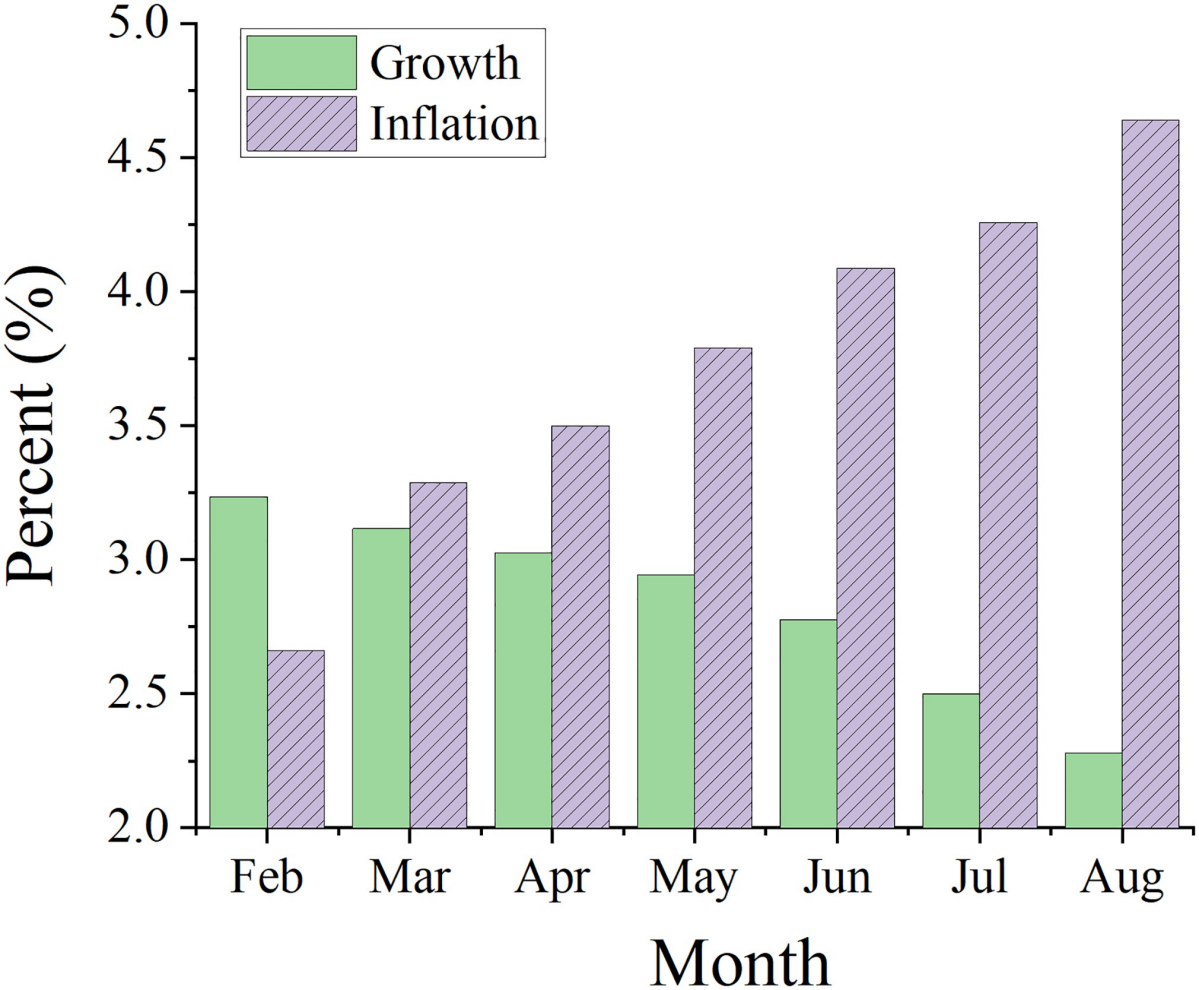
Expectations of global economic growth and inflation in 2023.

The rate of global development is already fast slowing and may continue to do so as more countries go through recessions. Emerging and developing economies will suffer long-term calamities if this pattern is permitted to continue. The markets most impacted by economic uncertainty are the stock market, followed by the money market, while the bond market is the market that is least impacted. Therefore, there will be risk transmission between interest rates and asset values as well as between short-term and long-term rates. There is a two-way feedback mechanism between domestic economic uncertainty and systemic financial risk in the process where systemic financial risk causes economic uncertainty and economic uncertainty reacts on systemic financial risk [20, 21]. Additionally, there is variation in the relationship between systemic financial risk, macroeconomic policy ambiguity, and uncertainty in economic cycles. Due to the stringent monetary and fiscal policies of different countries, these two variables may reinforce one another, tightening financial rules even more and impeding economic growth. Policymakers in emerging and developing economies need to be ready to deal with any spillover effects of internationally tightened policies. The projected pace of global economic growth and inflation for 2023 is shown in Fig 1.

**Fig 2:**
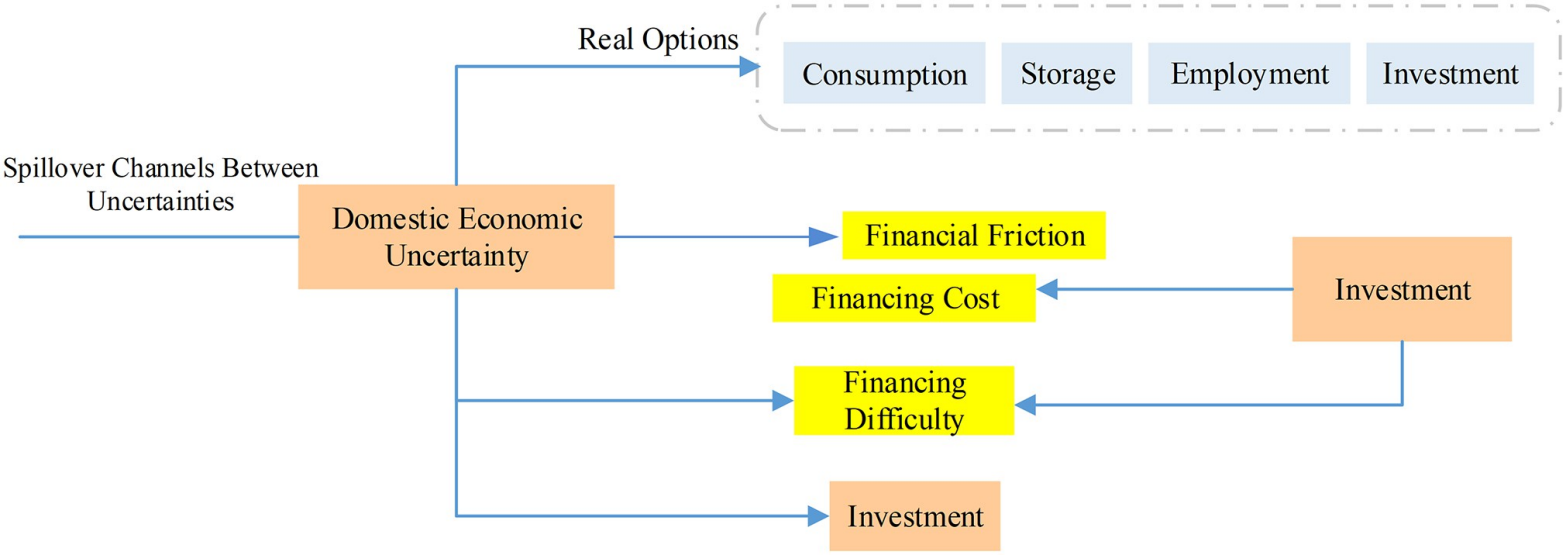
Spillover channels between external economic uncertainties.

The impact of uncertainty on the incomplete capital market causes the actual or perceived enterprise risk to increase, which in turn causes the expected probability of default risk or risk premium to increase, which in turn causes the expected return on investment to decrease, the cost of external financing to rise, and the willingness of investors to invest to decline, thereby reducing the investment scale [22]. Rising uncertainty will widen the gap in expected return on investment between businesses, which will then trigger a redistribution of capital among them. However, financial friction will delay this horizontal flow, which will eventually have a negative effect on the productivity and output of all factors. The difficulty of financing businesses grows due to the uncertain economic outlook, which in turn causes a decrease in investment and R&D. Policymakers will need to take decisive action to combat inflation, including loosening restrictions on the labor market, fostering global commodity supply and coordination, increasing food and energy supplies, supporting global trade networks, and cooperating to reduce tensions in the global supply chain. The short phase, followed by the weakest and most consistent effects over the intermediate period, shows the most pronounced and fluctuating effects of economic uncertainty on systemic financial risks. The pathways via which external economic uncertainty interact with one another are shown in Fig 2.

### 2.3 Constructing TVP-VAR model with stochastic volatility

The TVP-VAR model is a model based on the VAR model that implies the coefficient matrix and covariance matrix are time-varying, allowing the model to capture the process of changing economic structure over time [23–25]. The research on Vector Autoregression (VAR) model by researchers, especially in dynamic estimation, reveals the relationship among different quantiles. Quantile VAR model is a generalized VAR model, which can consider the dynamic relationship between variables under different quantiles by introducing conditional quantile function. De Luca et al. (2020) used the quantile vector autoregressive method based on copula to test the conditional quantile behavior of some assets in Eurostoxx50 relative to the quantile of the portfolio representing the market, and compared the results with the bivariate CAViaR model [26]. The results showed that copula method was very competitive and provides a time-varying model to better explain the value at risk, especially in terms of loss function. Chen et al. (2019) conducted bivariate volatility, value at risk and marginal expected gap based on Bayesian sampling scheme through adaptive Markov chain Monte Carlo method, so that all unknown model parameters and forecasts can be estimated statistically at the same time, which is helpful to jointly measure the downside tail risk of the industry [27].

Impulse response analysis is an important method to study the interaction between economic variables, which can help understand the impact or fluctuation of one economic variable on another. Impulse response analysis is usually combined with time series model. Impulse response function is a time series, which is used to describe the response of an economic variable to an external shock. The impulse response function shows the response degree of the analyzed economic variables to external shocks at different time points. Usually, the impact is expressed as an increase in standard deviation. VAR model is a multivariable time series model, which takes into account the interrelationships among multiple economic variables. In the VAR model, each variable is modeled as a linear combination at a certain time point in the past, and the impulse response function is used to measure the response of these variables to external shocks. Stolbov et al. (2021) used the mixed econometric method to capture the nonlinear nature of macro-financial links of 16 European economies from January 1997 to December 2019 by establishing different quantiles of data series and estimating impulse response according to smooth local prediction [28]. Yang et al. (2023) constructed a sovereign default network by analyzing the connectivity of sovereign credit default swap market and using high-dimensional vector autoregressive [29]. Yang (2023) studied the influence of network centrality on the special sovereign risk during the oil price bubble. The research results emphasized the importance of sovereign default network in the transmission of special sovereign risk and systemic sovereign risk in bubble events [30]. Yang and Cui et al. (2023) used the mixed data sampling conditional value method of risk quantile regression, combined with data of different frequencies, proved the possibility of risk spillover in external markets, and predicted domestic macroeconomic shocks [31]. Yang (2019) studied the causal relationship between the uncertainty of economic policy and the oil price shock and the cross-time scale connection. The wavelet method was incorporated into the structural vector autoregressive framework, and it was found that the crude oil price was like the receiver of economic policy uncertainty information regardless of the time scale [32].

The research of these documents provides valuable discussion for revealing the dynamic relationship among different quantile VAR models. By considering the conditional distribution characteristics under different quantiles, quantile VAR model can reflect the relationship among variables more accurately, especially in the face of different risk levels and shocks. This helps people to better understand the heterogeneity and nonlinear characteristics of the economic system and the linkage between different markets, industries, or economic variables. It is worth noting that the literature mentioned above is only a small part of the research on quantile VAR model, and there are many other literatures and methods that can be further explored. The results and discussions of these studies are very important for people to deeply understand the quantile VAR model and the relationship among different quantiles, and help people to make more accurate predictions and decisions in the macroeconomic and financial fields. The goal of developing a VAR model is to validate the optimal lag order before performing a Granger causality test. The Granger causality test is used to determine the impact order of economic variables in time series. In the Granger causality test, people must select the lag order, which in this case is the lag order verified when establishing the VAR model. It should be mentioned that for the Granger Causality test, stable data are required. If it is unstable, it can be processed differently, but it is important to take into account the fiscal implications. Di Giovanni et al. (2022) studied the transmission of global financial cycle to domestic credit market conditions in Turkey’s large emerging markets from 2003 to 2013 [13], in which credit risk refers to the potential risk that borrowers or debtors cannot fulfill their repayment obligations on time or in full in lending and capital provision activities. It involves the possibility of the borrower’s default, that is, the borrower cannot repay the principal and interest, which leads to the creditor’s failure to get the due return. Credit risk is usually related to the debtor’s solvency, credit rating, default probability and other factors. Bankruptcy means that an enterprise or individual is unable to pay off debts, is insolvent and is sentenced to bankruptcy by the court. Bankruptcy is an extreme case of credit risk, which means that the debtor cannot perform his debts and needs to restructure or liquidate his debts through legal procedures. Bankruptcy involves the debtor’s capital flow, which may lead to creditors’ inability to fully obtain their claims. The causal relationship between institutional quality and credit risk and bankruptcy may be two-way. On the one hand, low institutional quality may increase the credit risk and bankruptcy risk faced by financial institutions. Factors such as inefficient supervision, corruption and imperfect legal system may weaken the capital adequacy and risk management ability of financial institutions and increase the possibility of credit default and bankruptcy. On the other hand, credit risk and bankruptcy events may also have an impact on the quality of institutions. Large-scale credit default and bankruptcy may undermine the stability of the financial system, trigger systemic financial risks, and weaken the performance of the overall economy. This further highlights the importance of institutional quality, including strong supervision, sound risk management and appropriate legal framework to deal with credit risks and bankruptcy incidents. Internationally, there is no unified consensus on the differences between credit risk and bankruptcy concepts. The definition of these concepts may be different due to differences in countries, industries, and academia. However, international research and practice generally recognize the close relationship between credit risk and bankruptcy, and pay attention to their impact on financial stability and economic development. Therefore, when studying the causal relationship between institutional quality, credit risk and bankruptcy, people should consider the differences between credit risk and bankruptcy concepts to ensure the accuracy and reliability of the research. The time-varying parameter VAR model can also be split into small-scale and large-scale VAR models based on the quantity of variables. The small-scale time-varying parameter VAR model is one of them and is referred to as TVP-VAR model [33–35].

When analyzing the spillover effect of American economic uncertainty on China’s macro-economy, and constructing the corresponding TVP-VAR model, this paper takes into account the nonlinear characteristics of macro-economy caused by institutional changes, financial crisis and other factors, so it is necessary to construct a TVP-VAR model with random volatility to explain the results of the nonlinear model. Volatility index is an important index to measure the level of market risk and uncertainty, which can provide information about the expected volatility of the market. The inclusion of the volatility index can make the study more comprehensively capture the impact of American economic fluctuations on China’s macro-economy. The dynamic relationship and interaction between the volatility index and other economic variables can be analyzed by incorporating them into the VAR model. This is helpful to evaluate the impact of the fluctuation of American economy more accurately on China’s macro-economy, and reveal the potential international spillover effect. The analysis of volatility index can provide a deeper understanding of market expectation, risk preference and market sentiment, which is very important to explain how the US economic fluctuations are transmitted to China’s macro-economy. Meanwhile, it can also help to predict and monitor potential risks and uncertainties, and help to formulate corresponding policies and risk management strategies. Therefore, the inclusion of the VIX in the VAR system can provide a more comprehensive, accurate, and in-depth analysis to reveal the international spillover effect of the United States on China. VIX measures market participants’ expectations of future market volatility. When VIX rises, it usually means that market participants are worried about future uncertainties and risks. Therefore, the inclusion of VIX index in the VAR model can help predict the changes of market risks and how these risks are transmitted to other financial and macroeconomic variables. The calculation method of volatility index varies from index to index, but it usually involves a model based on option price and implied volatility. These models consider the prices of different option contracts to estimate the expectations of market participants for future volatility. Eq 1 shows the calculation expression:

$$Historical\;Volatility=\sqrt{\frac{1}{N-1}\sum_{i=1}^{N}{\left(R_{i}-\bar{R}\right)}^{2}}\times \sqrt{P}$$ (1)

*N* is the number of trading days during the observation period, *R*_*i*_ is the yield of the *i*th trading day, $\bar{R}$ is the average of the yield, and *P* is the number of trading days per year.

A *k*-dimensional structured linear VAR model with lag time *p* is set:

$$Ay_{i}=\sum_{j=1}^{p}F_{j}y_{t-j}+u,t=p+1,p+2,\ldots ,T$$ (2)

*y* represents the observable endogenous variable of *k* × 1. *F* represents the coefficient matrix of *k* × 1. *u* is the error term. *T* is the number of samples.

If the synchronization relation matrix A among variables is a lower triangular matrix, the follows can be obtained:

$$A=\left(\begin{array}{llll} 1 & 0 & \ldots & 0\\ a_{21} & 1 & \ldots & 0\\ \ldots & & & \\ a_{k1} & \ldots & a_{k,k-1} & 1 \end{array}\right)$$ (3)

A simplified linear VAR model can be obtained after operation processing, which is expressed as:

$$y_{t}=\sum_{j=1}^{p}B_{j}y_{t-j}+A^{-1}\sum \varepsilon_{t},\varepsilon \sim N\left(0,I_{k}\right)$$ (4)

$$B_{j}=A^{-1}F_{j}$$ (5)

$$\sum =\left(\begin{array}{llll} \sigma_{1} & 0 & \ldots & 0\\ 0 & \sigma_{2} & \ldots & 0\\ \ldots & & & \\ 0 & \ldots & 0 & \sigma_{k} \end{array}\right)$$ (6)

*σ*_*j*_ represents the standard deviation of structural shock.

In many cases, the process of generating economic data has the impact of drift coefficient and random fluctuation. If this is the case, using the model with time-varying coefficient but constant fluctuation will cause a problem, that is, the estimated time-varying coefficient may be biased because the possible change of fluctuation in disturbance is ignored. In order to avoid this problem, TVP-VAR model assumes random volatility [36, 37]. In order to upgrade the TVP-VAR model to a TVP-VAR model with random volatility, it is necessary to set the residual standard deviation matrix ∑ to change with time, and the optimized model can be expressed as:

$$y_{t}=\sum_{j=1}^{p}B_{j}y_{t-j}+A^{-1}\sum t\varepsilon_{t},\varepsilon \sim N\left(0,I_{k}\right)$$ (7)

$$A_{t}y_{t}=X_{t}\beta_{t}+\varepsilon_{t}$$ (8)

$$\beta_{t}=\beta_{t-1}+\eta$$ (9)

The vector *β*_*t*_ represents the *B* vector formed by the accumulation of elements in the coefficient matrix *k*^2^ *s* × 1.

### 2.4 Model estimation based on MCMC-Gibbs sampling method

The estimation and prediction of the TVP-VAR model with random volatility built in this paper will be very challenging due to the model’s high level of parameterization. The TVP-VAR model is linear at each time point, but overall, the model is extremely nonlinear. Therefore, the likelihood function is challenging to handle, the random volatility makes the estimation difficult, and the conventional estimation methods, such as the least square method and maximum likelihood method, cannot estimate the model effectively. Based on the Gibbs sampling technique, the MCMC method will be used in this paper to estimate the model for Bayesian inference. For time-varying parameter models in high-dimensional state space, the MCMC-Gibbs sampling technique is appropriate [38–40]. Using MCMC-Gibbs method to analyze the international spillover effect of American economy on China’s macro-economy can overcome the challenge of high-dimensional state space, realize Bayesian inference of model parameters, and effectively obtain the posterior distribution of parameters through Markov chain sampling and Gibbs sampling technology. MCMC-Gibbs method makes Bayesian inference by sampling technology and estimates the parameters of the model. The international spillover effect of American economy on China’s macro-economy can be analyzed by MCMC-Gibbs method, and the posterior distribution of parameters can be obtained based on the existing observation data and prior knowledge to better understand the influence of American economy on China’s economy. In order to understand the international spillover effect of American economy on China’s macro-economy, MCMC method samples the parameter space by constructing Markov chain, and generates a series of parameter samples to approximately represent the posterior distribution of parameters, which can generate samples close to the posterior distribution of parameters. In the process of Gibbs sampling, only one parameter in the model is updated in each iteration, while the other parameters remain unchanged. This step-by-step iterative method can effectively sample from posterior distribution and improve the efficiency of parameter estimation.

**Fig 3:**
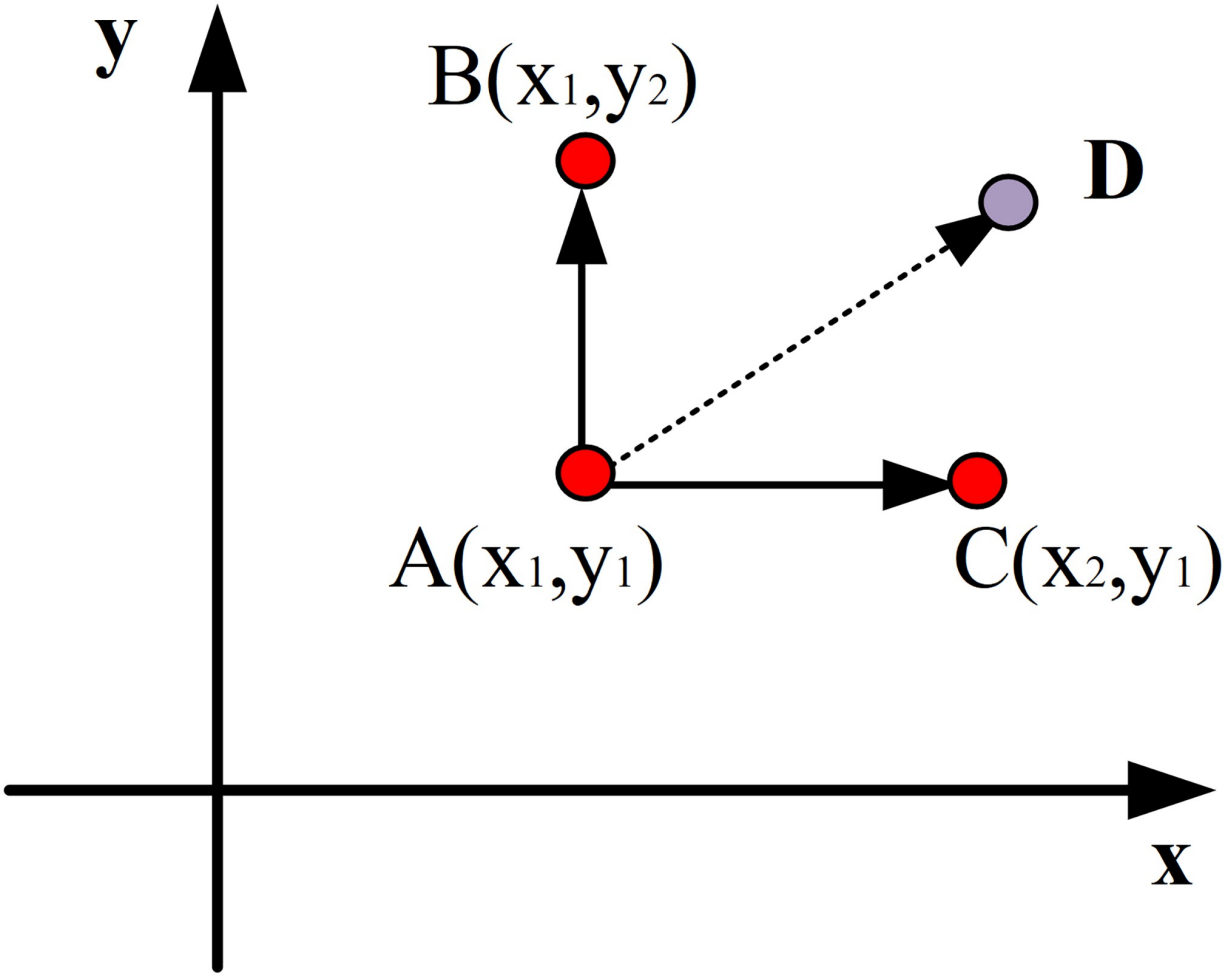
Construction of Markov chain transfer matrix on plane.

The fundamental idea behind the MCMC technique is to create a Markov chain and convert the parameter distribution into a Markov stationary distribution. The MCMC method can effectively simulate the samples that match the posterior distribution for some posterior distributions without a specific expression, allowing for the estimation of parameter values. Table 1 displays the particular MCMC method operational flow. Fig 3 depicts the Markov chain transfer matrix’s creation on a plane.

**Table 1:** Specific operation flow of MCMC method.

MCMC sampling algorithm
**1:** Initialize initial state *X*_0_ = *x*_0_ of Markov chain
**2:** For *t* = 0, 1, 2…, cycle the following process to sample:
At the *t* time, the Markov chain state is *X*_*t*_ = *x*_*t*_, and *y* ~ *q*(*x|x*_*t*_) is sampled
Sample *u*~*Uniform*[0, 1] from uniform distribution
If *u* < *α*(*x*_t_, *y*) = *p*(*y*)*q*(*x*_*t*_*|y*) is accepted, transfer *x*_*t*_ → *y*, that is, *X*_*t*+1_ = *y*
If not, the transfer is not accepted, that is, *X*_*t*+1_ = *x*_*t*_

Gibbs sampling is a sampling method for high-dimensional distribution. Firstly, a target distribution *p*(*x*) is given, in which the first criterion is that the conditional distribution of each variable has an analytical expression based on the joint distribution of all other variables. Formally, if the target distribution *p*(*x*) is *D* -dimensional, there must be *D* independent expressions:

$$p\left(x_{i}\left|x_{\prime },x_{2},\ldots ,x_{i+1},x_{i-1},\ldots x_{D}\right.\right)=p\left(x_{i}\left|x_{j}\right.\right)$$ (10)

Each expression defines the probability of the *i* -th dimension when other *j* -dimensional values are known. Having a conditional distribution of each variable means that there is no need to propose a distribution or accept/reject criteria like the Metropolis Hastings algorithm. Therefore, when other variables are fixed, we can simply sample from each condition.

The working method of Gibbs sampling is similar to that of the component Metropolis Hastings algorithm. Except that the proposed distribution of each dimension is banned, the value of this dimension is selected simply according to the conditional distribution corresponding to variables for the accepted or rejected proposed sampling [41]. Similar to the component Metropolis Hastings algorithm, it passes through each variable in turn and samples it when other variables are fixed. Gibbs sampling steps are roughly as follows: (1) Set *t* = 0. (2) Generating an initial state *x*^(0)^ ~ *π*^(0)^. (3) Repeat until *t* = *M* (for each dimension *i*, get *x*_*i*_ from *p*(*x*_*i*_|*x*′, *x*_2_,…, *x*_*i*+1_, *x*_*i*-1_,… *x*_*D*_)).

### 2.5 Data selection and model application

Details of MCMC-Gibbs model specification: First, the model used to analyze the international spillover effect of American economy on China’s macro-economy is determined. The time-varying parameter-stochastic volatility-vector autoregressive (TVP-VAR) model is adopted. The model considers the impact of uncertainty on the economy and introduces stochastic volatility to better describe the changing characteristics of data. Secondly, MCMC-Gibbs method will be used to estimate the model parameters. This method samples the parameter space by constructing Markov chain and generates posterior distribution samples of parameters. Specifically, for each parameter in the TVP-VAR model, Gibbs sampling technique is used for iterative updating. At each iteration, a parameter in the model is updated according to the known parameters and data by conditional distribution sampling method. In order to make Bayesian inference, it is necessary to set the prior distribution of parameters. The prior distribution reflects the previous knowledge or assumptions about the parameters. In MCMC-Gibbs model, it is necessary to assign a prior distribution to each parameter and set its super parameter according to prior knowledge or experience. These prior distributions will be combined with the data to obtain the posterior distribution of parameters. In the MCMC-Gibbs model specification, the economic data samples of China and the United States from January 2001 to January 2022 in WIND database and FRED database are used. These data will be the basis of model estimation and analysis. Finally, when MCMC-Gibbs sampling is carried out, convergence diagnosis is carried out to evaluate the quality of sampling results. This is achieved by checking the autocorrelation of samples, the stability of parameter values during iteration and the mixing degree of posterior distribution. Economic ambiguity in the United States and macroeconomic factors in China are chosen as the main variables for this paper. In combination with Fofack et al. (2020)’s research on evaluating the surge in capital flows to developing and emerging market economies caused by the experience of the Federal Reserve’s quantitative easing [42], it is discovered that GDP, inflation, and interest rates are the primary factors that make up macroeconomic communication, and that the capital flow channel largely determines the cross-border spillover impact of economic uncertainty. The effects of exchange rate, American policy and business cycle on China’s macro-economy can be discussed from many aspects. First, exchange rate is the exchange rate between two countries’ currencies, which has an important impact on China’s macro-economy. The change of exchange rate can directly affect China’s export competitiveness. If China’s currency depreciates, the price of export commodities will become more competitive in the international market, which will help to increase export volume and export income. Exchange rate changes will also have an impact on imports. If China’s currency appreciates, the prices of imported goods will become cheaper, which may lead domestic enterprises to be more inclined to import, thus impacting domestic industries. Secondly, the United States is one of the largest economies in the world, and its policies have a direct and indirect impact on China’s macro-economy. Changes in US trade policies (such as tariffs, trade disputes, etc.) will have an impact on China’s exports and imports, and then have an impact on China’s macro-economy. Protectionist measures may lead to a decrease in China’s exports, which will have a negative impact on China’s manufacturing and related industries. The adjustment of US monetary policy will have an impact on global financial markets and exchange rates, including China. The decision of raising interest rates or lowering interest rates in the United States will have an impact on global capital flows and money supply, and then on China’s monetary policy and economic and financial environment. Finally, the economic development of the United States and China is often in an interrelated business cycle. Business cycle fluctuations in the United States will have spillover effects on China’s macro-economy. For example, the economic recession in the United States may lead to a decline in demand and exports, which will have a negative impact on China’s economic growth and employment. Business cycle fluctuations in the United States will also be transmitted to China through financial markets. The turbulence of global financial market and the change of risk preference will cause international capital flow and exchange rate fluctuation, which will have an impact on China’s financial market and capital flow. In order to determine whether the uncertainty of the American economy will have an impact on China’s economy through capital flow channels, this paper includes the stock price and the actual effective exchange rate of the RMB. Table 2 displays the findings of the stationarity tests for each variable.

**Table 2:** Variables and their stationarity test results.

Variable name	Variable symbol	ADF test results
American economic uncertainty	USUNC	-3.222**
Economic uncertainty in China	CNUNC	-5.425***
Investment	INV	-0.343
Consumption	CONS	-5.570***
Total import	IMP	-20.965***
Total export	EXP	-15.282***
GDP	GDP	-5.623***
Price level	PRICES	-4.981***
Short-term interest	RATES	-5.289***
Price of stock	SHARE	-5.349***

Note:

*** and ** mean rejecting the original hypothesis at the significance level of 1% and 5% respectively.

In Table 2, both the US Economic Uncertainty (USUNC) and the China Economic Uncertainty (CNUNC) rejected the original hypothesis in the ADF test, that is, their difference sequences are stationary. This shows that the uncertainty of American economy is related to the uncertainty of China economy to some extent. In addition, at the significance level of 1% and 5%, consumption (CONS), total import (IMP), total export (EXP), GDP, price level (PRICES), short-term interest rate (RATES) and stock price (SHARE) all rejected the original hypothesis in ADF test. This shows that there is a correlation between investment and consumption, between total imports and total exports, between GDP and price level, and between short-term interest and stock prices. These results show that there is a significant correlation among these variables. The USUNC has a significant impact on China’s macro-economy. In particular, China’s consumption, import, export, GDP and price level are affected by USUNC. The impulse response function is computed using the Cholesky decomposition method. The model’s degree of freedom will decrease with increasing lag time. The lag period of the benchmark model and the model with the corresponding variables in this paper is set to 3, which is sufficient to capture the model and possible economic trends while maintaining the model’s freedom and producing unrelated residual sequences.

## 3. Results and discussions

### 3.1 Analysis of the results of economic uncertainty construction in the United States and China

**Fig 4:**
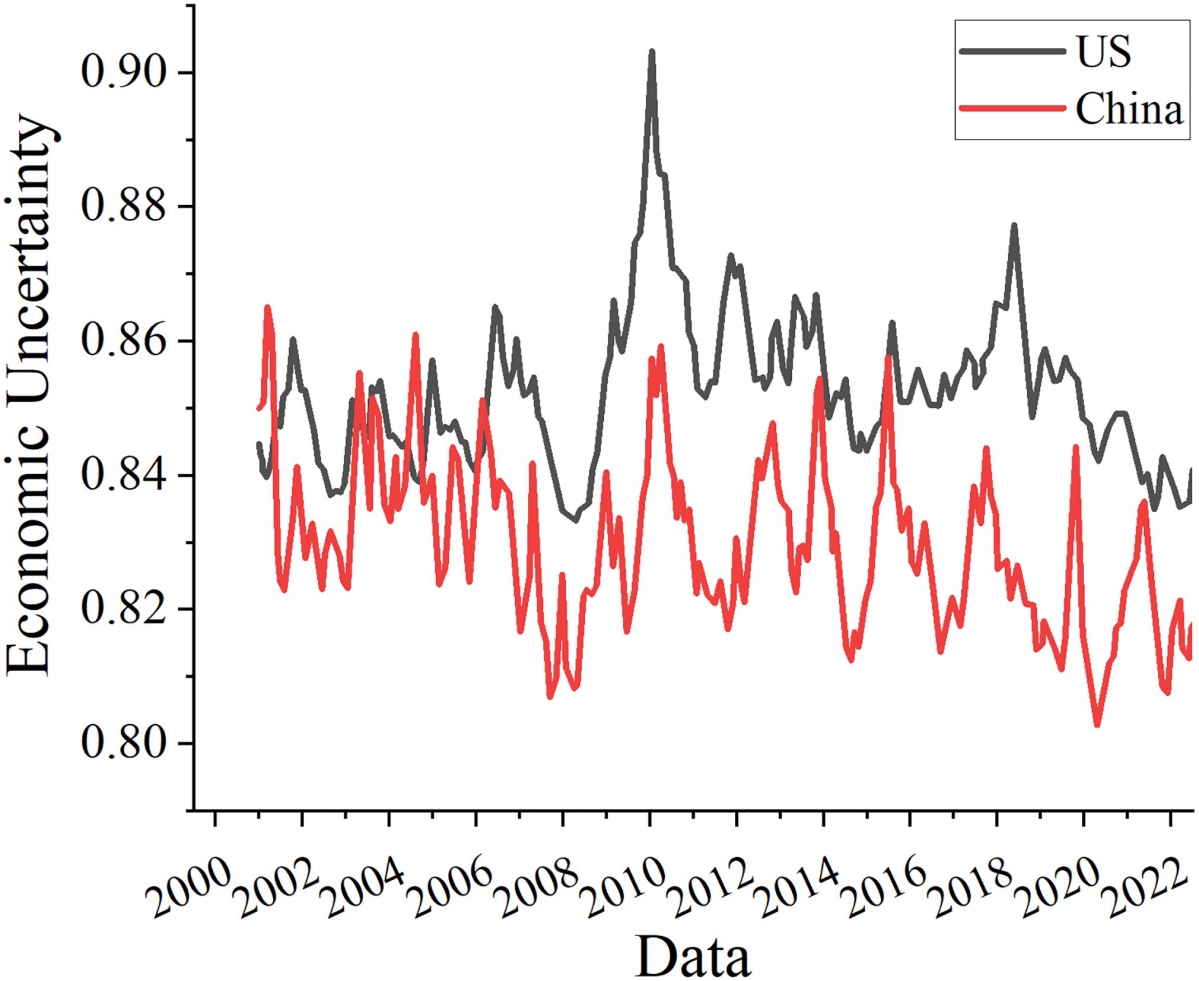
Estimates of economic uncertainty in the United States and China.

According to the MCMC-Gibb model, Fig 4 depicts how unpredictable the US and Chinese economies are. It was discovered that the American economy’s estimated level of uncertainty peaked for the first time at the end of 2001, which may have been influenced by the panic that the "9–11" disaster produced in the country. The American economy’s level of uncertainty then began to diminish until the Gulf War in 2003, when it once more rose. Then, as a result of the financial crisis, there was a substantial increase in economic unpredictability in the US, with two peaks occurring in 2008 and 2009. After 2009, the US economy’s level of uncertainty gradually decreased, with the exception of a minor recovery in 2016, which may have been influenced by the US presidential election. The model developed in this paper’s estimation of U.S. economic uncertainty has clearly increased during the significant economic policy events, indicating that the U.S. economic uncertainty developed in this paper is reasonable.

Nonetheless, China’s economic unpredictability has faced a number of extreme crises, including those at the end of 2001, the start of 2009, the start of 2012, and in February 2020. Since China joined the World Trade Organization in November 2001, it has been simpler for external uncertainties to have an impact on China. Beginning in 2009, the negative impacts of the world financial crisis started to become apparent, external demand decreased, and there was a lot of uncertainty about the state of the world economy. Domestic overcapacity resulted in greater economic uncertainty at the start of 2012 because of insufficient domestic demand and declining foreign demand. Trump’s election as US president in 2017 also contributed to China’s extremely high level of economic unpredictability. The COVID-19 epidemic started in China in February 2020. It significantly harmed the country’s economy and caused a sharp increase in economic unpredictability in China.

**Fig 5:**
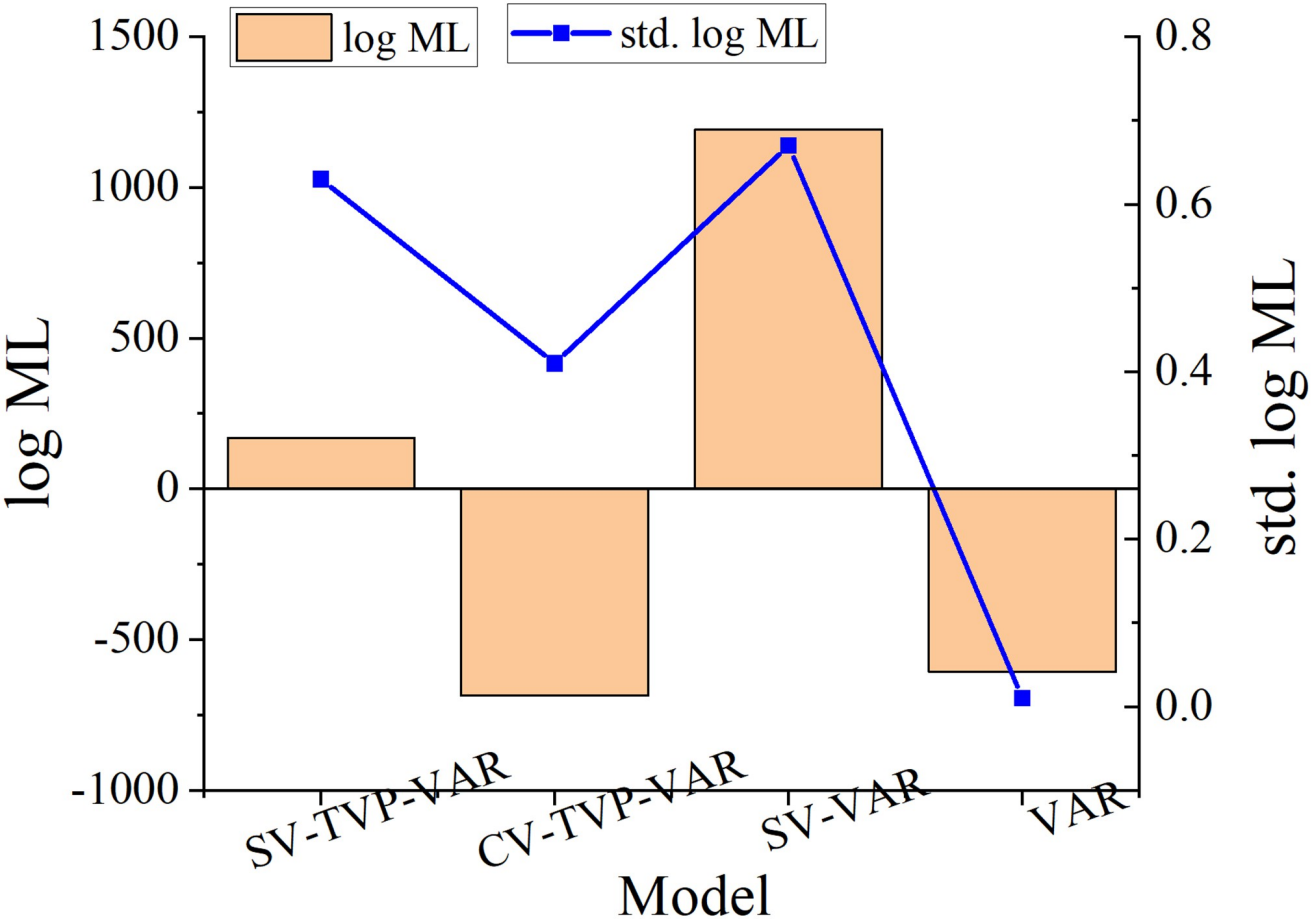
Logarithmic marginal likelihood of different estimation models.

The TVP-VAR model with random volatility (SV-TVP-VAR) proposed in this paper is compared with other estimation models, namely the VAR model with random volatility (SV-VAR), the TVP-VAR model with constant volatility (CV-TVP-VAR), and the standardized unlimited VAR model, in order to confirm the validity of the model proposed in this study for time-varying settings. (VAR). The logarithmic marginal likelihood (log ML) and its associated standard deviation (std.log ML) of various models are computed, and the precise results are displayed in Fig 5. The VARs lag period of all the comparison models is set to 3, and the results are displayed in Fig 5.

### 3.2 Time-averaged cumulative impulse response analysis

**Fig 6:**
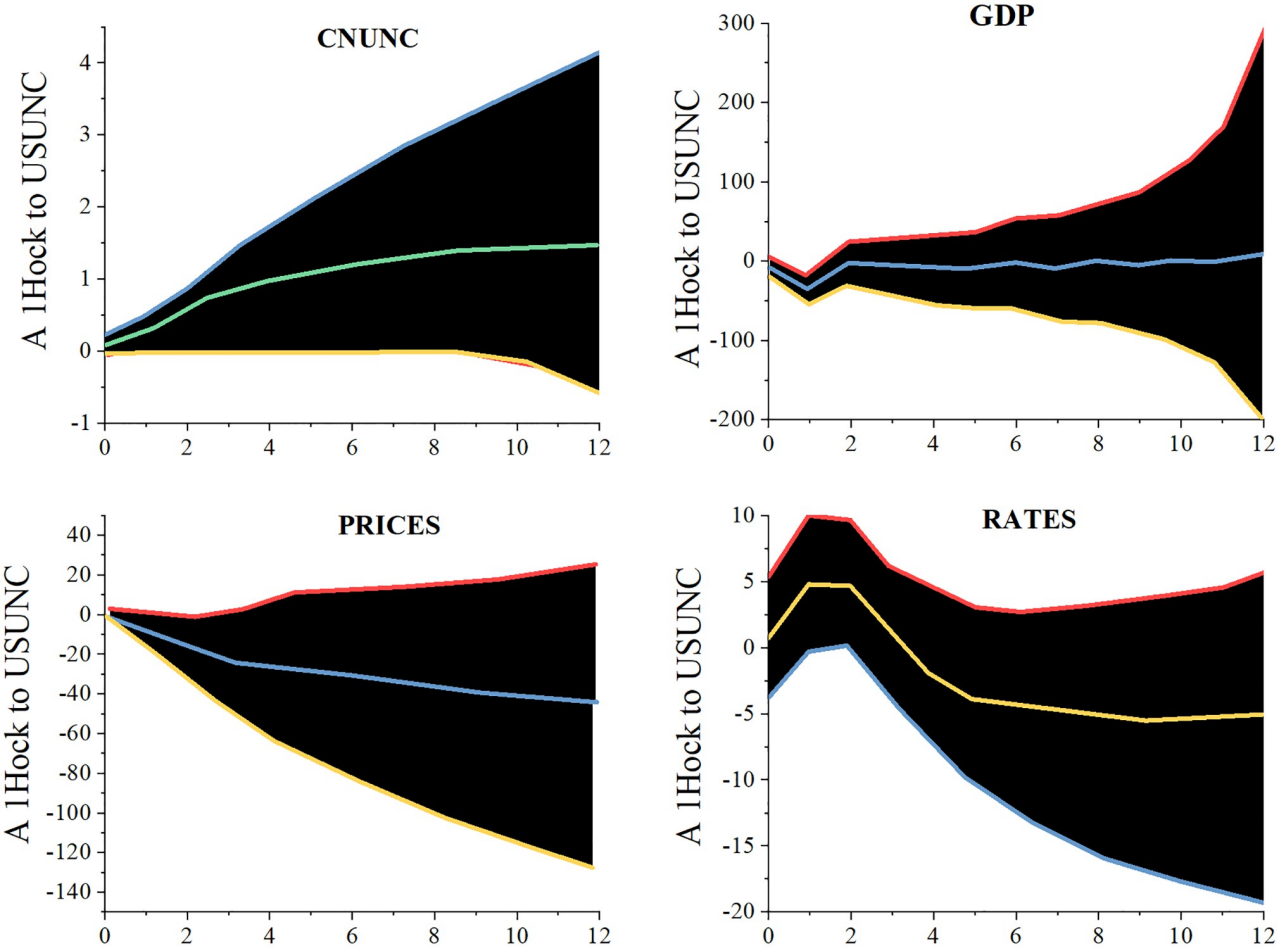
Time-averaged cumulative impulse response function.

In reaction to the uncertain growth of the US economy, Fig 6 shows the time-averaged responses of China’s GDP, price level, and short-term interest rate. The shaded area in the figure represents the 68% highest probability density interval, which is calculated using the 16% and 84% percentiles of the posterior estimated value of the impulse response function, and the straight line in the figure represents the median estimated value of the impulse response function. According to the research, economic ambiguity in the United States will negatively affect China’s GDP, price level, and short-term interest rate overall, while economic uncertainty in China will have the opposite effect. China’s economic uncertainty will exhibit a significant growth trend after the effect of the United States’ economic uncertainty growth, and from the eighth month after the impact, the response will steadily stabilize.

**Fig 7:**
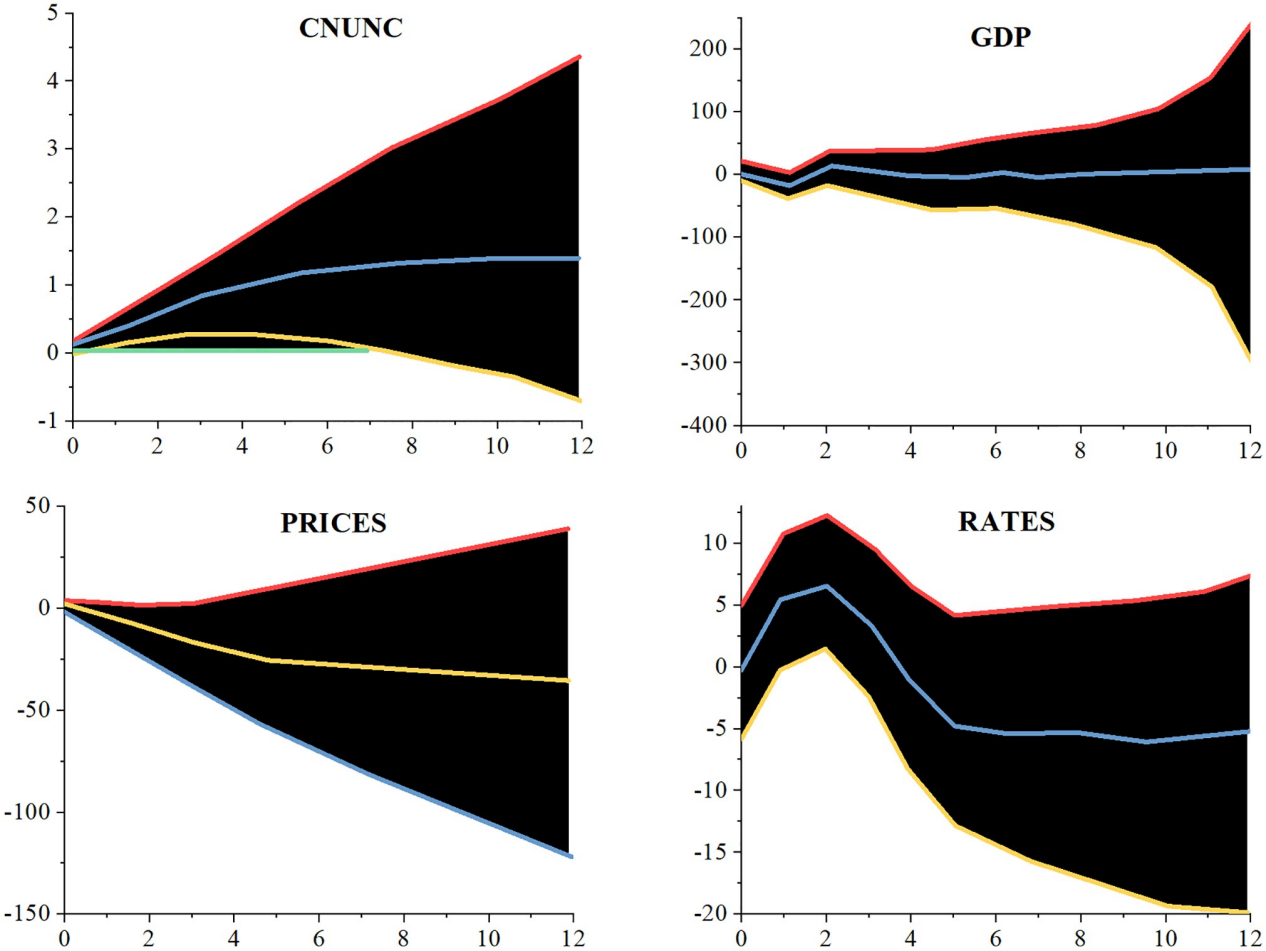
Cumulative impulse response function of time average under capital flow channel.

**Fig 8:**
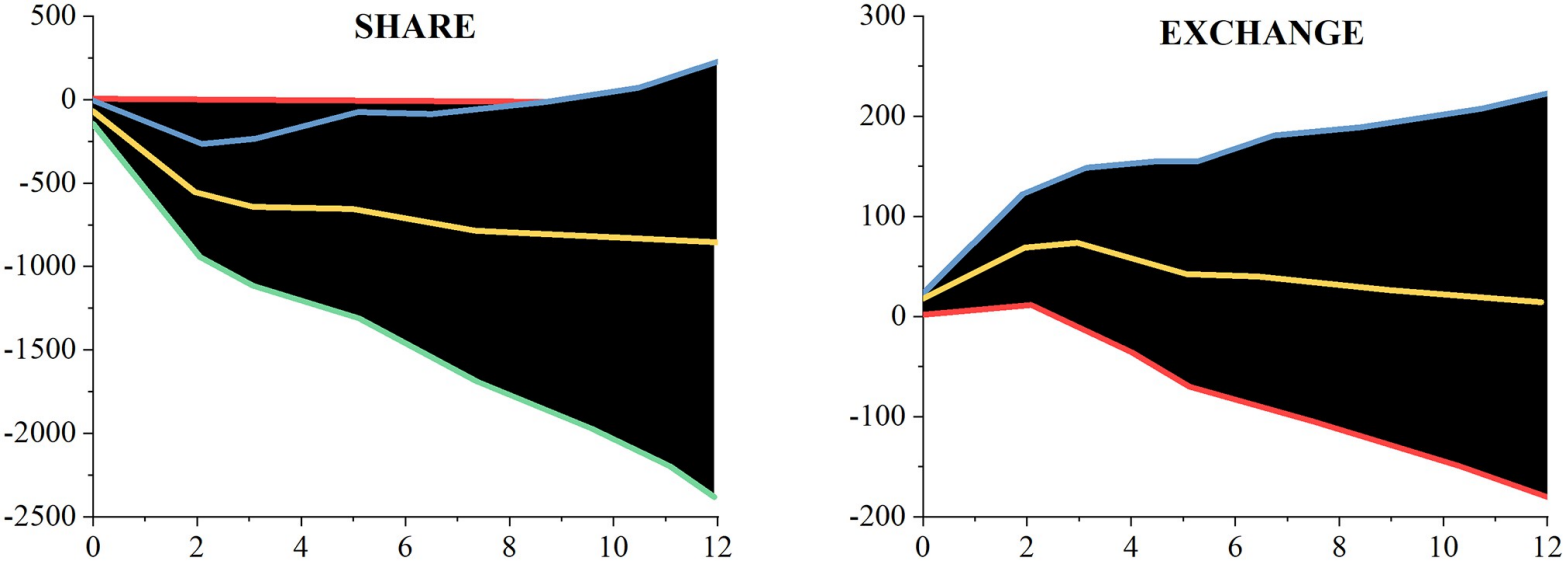
Cumulative impulse response function of time average with stock price in capital flow channel.

Fig 7 depicts the time-average cumulative impulse response curve for the capital flow channel of the estimate model. Fig 8 displays the time-averaged cumulative impulse response function diagram of the model together with the stock price. It demonstrates that China’s stock price fell sharply following the bad impacts caused by the unstable American economy until the decrease stopped being apparent nine months after the negative effects. In contrast, the real effective exchange rate of the RMB showed a positive response throughout the response period, however it was not until the first two months after the shock that it really started to show. In other words, the US economy’s cyclicality was to blame for the RMB’s decline. The responses of the other variables largely match those of the baseline model: GDP and price levels decline, economic uncertainty in China continues to rise after the impact, and short-term interest rates begin to decline after briefly rising in the first three months after the impact. According to the findings of Hu et al. [43], who attributed the decrease in China’s stock price to the American market’s undeniable domination of the global stock market, the decline in China’s stock price was caused by the uncertainty shock in the United States.

This empirical study demonstrates how the detrimental effects of U.S. economic uncertainty have altered significantly over time in relation to China’s macroeconomic variables. During the global financial crisis, the American economy’s uncertainty is, on the whole, more severe, lasted longer, and has a greater overall effect. Economic uncertainty in China is prolonged during the financial crisis by the intensification of the effects of economic unrest in the United States, which includes a steep decline in GDP, the level of prices, and the short-term interest rate. The American financial crisis has a long-lasting negative impact on China’s stock market, but as time goes on, its evolution is marked by the Chinese government’s quick response to external shocks and the implementation of stimulus measures, which helps the market gradually recover after the financial crisis reaches its lowest point. Through financial channels, the economic instability in the United States will have an impact on China’s economy due to the depreciation of the RMB. No nation can handle the problem by itself.

It emphasizes how China’s economy is more vulnerable to the effects of global economic uncertainty during turbulent and high-uncertainty periods, and that this susceptibility is predominantly communicated through channels of capital flow. As a result, China should, in the face of external economic shocks, not only take preventative measures but also collaborate with other countries to safeguard its political, economic, and cultural interests, forge a sense of shared destiny, and cooperate to protect and guard against the effects of financial risks. Additionally, the central bank needs to take the required actions to maintain the exchange rate to decrease the RMB’s potential short-term volatility in response to outside shocks.

## 4. Conclusions

The dynamic relationship between the macroeconomic conditions in China and the United States is examined and tested using the TVP-VAR model, and the model is estimated using the Gibbs sampling technique based on MCMC. The time-varying dynamics of the relationship between the economic uncertainty of the United States and China’s macro-economy are empirically tested using the economic data samples of China and the United States from January 2001 to January 2022, which are collected from the WIND database and the FRED database, respectively. This paper illustrates the channels through which American economic uncertainty is transmitted to China’s economy and examines more thorough responses of China’s macro-economy to external shocks. It is based on a theoretical overview of cross-border channels of external economic uncertainty. Since this paper only focuses on the macroeconomic level, future research can examine the effects of microeconomic factors that have spillover effects, such as the effect of foreign economic uncertainty on investor sentiment.

## Supporting information

S1 Data (XLSX)

## References

[1] Deng Q, Xiao W, Yan H. The spillover effects of US monetary policy normalization on the BRICS based on panel VAR Model[J]. Journal of Mathematics, 2022, 2022: 1–9.

[2] Ouyang Z, Dou Z, Wei L, et al. Nonlinear spillover effect of US monetary policy uncertainty on China’s systematic financial risks[J]. Journal of Business Economics and Management, 2022, 23(2): 364–381–364–381.

[3] Huang N, Huang N, Wang Y. US economic policy uncertainty on Chinese economy: industry level analysis[J]. Applied Economics Letters, 2020, 27(10): 789–802.

[4] Sznajderska A, Kapuściński M. Macroeconomic spillover effects of the Chinese economy[J]. Review of International Economics, 2020, 28(4): 992–1019. doi: 10.1111/roie.12479 32836997 PMC7264539

[5] Abbas G, Wang S. Does macroeconomic uncertainty really matter in predicting stock market behavior? A comparative study on China and USA[J]. China Finance Review International, 2020, 10(4): 393–427.

[6] Yang J, Yu Z, Ma J. China’s financial network with international spillovers: A first look[J]. Pacific-Basin Finance Journal, 2019, 58: 101222.

[7] Wang Z, Li Y, He F. Asymmetric volatility spillovers between economic policy uncertainty and stock markets: Evidence from China[J]. Research in International Business and Finance, 2020, 53: 101233.

[8] Li Z, Hou J, Zhang J. Spillover effect of crude oil futures market: an empirical research from emerging market[J]. Sustainable Energy Technologies and Assessments, 2022, 53: 102695.

[9] He F, Ma F, Wang Z, et al. Asymmetric volatility spillover between oil-importing and oil-exporting countries’ economic policy uncertainty and China’s energy sector[J]. International Review of Financial Analysis, 2021, 75: 101739.

[10] Gabauer D, Gupta R. Spillovers across macroeconomic, financial and real estate uncertainties: a time-varying approach[J]. Structural Change and Economic Dynamics, 2020, 52: 167–173.

[11] Li Y, Zhuang X, Wang J, et al. Analysis of the impact of Sino-US trade friction on China’s stock market based on complex networks[J]. The North American Journal of Economics and Finance, 2020, 52: 101185.

[12] Mohaddes K, Raissi M, Sarangi N. Macroeconomic effects of global shocks in the GCC: Evidence from Saudi Arabia[J]. Middle East Development Journal, 2022, 14(2): 219–239.

[13] Di Giovanni J, Kalemli-Özcan Ş, Ulu MF, et al. International spillovers and local credit cycles[J]. The Review of Economic Studies, 2022, 89(2): 733–773.

[14] Abbas G, Hammoudeh S, Shahzad S J H, et al. Return and volatility connectedness between stock markets and macroeconomic factors in the G-7 countries[J]. Journal of Systems Science and Systems Engineering, 2019, 28: 1–36.

[15] Wu M, Zhu Z. The Volatility Spillover Effect Between the International Crude Oil Futures Price and China¡¯ s Stock Market-Multivariate BEKK-GARCH Model Based on Wavelet Multiresolution[J]. International Journal of Financial Research, 2019, 10(4): 84–89.

[16] Cepni O, Guney IE, Swanson NR. Forecasting and nowcasting emerging market GDP growth rates: The role of latent global economic policy uncertainty and macroeconomic data surprise factors[J]. Journal of Forecasting, 2020, 39(1): 18–36.

[17] McIver RP, Kang SH. Financial crises and the dynamics of the spillovers between the US and BRICS stock markets[J]. Research in International Business and Finance, 2020, 54: 101276.

[18] Wei Y, Qin S, Li X, et al. Oil price fluctuation, stock market and macroeconomic fundamentals: Evidence from China before and after the financial crisis[J]. Finance Research Letters, 2019, 30: 23–29.

[19] Jiang C, Li Y, Xu Q, et al. Measuring risk spillovers from multiple developed stock markets to China: A vine-copula-GARCH-MIDAS model[J]. International Review of Economics & Finance, 2021, 75: 386–398.

[20] Rout SK, Mallick H. International spillovers of interest rate shocks: An empirical analysis[J]. Asian Journal of Empirical Research, 2020, 10(10): 215–222.

[21] Balli F, Hasan M, Ozer-Balli H, et al. Why do US uncertainties drive stock market spillovers? International evidence[J]. International Review of Economics & Finance, 2021, 76: 288–301.

[22] Siklos PL. The macroeconomic response to real and financial factors, commodity prices, and monetary policy: International evidence[J]. Economic Systems, 2021, 45(1): 100850.

[23] Tumala MM, Salisu AA, Atoi NV, et al. International monetary policy spillovers to emerging economies in Sub-Saharan Africa: A global VAR analysis[J]. Scientific African, 2021, 14: e00976.

[24] Bi Y, Xin G. Analysis of economic shock effects between China and ASEAN: An empirical study based on multinational VAR[J]. Emerging Markets Finance and Trade, 2021, 57(8): 2290–2306.

[25] Ren Y, Guo Q, Zhu H, et al. The effects of economic policy uncertainty on China’s economy: evidence from time-varying parameter FAVAR[J]. Applied Economics, 2020, 52(29): 3167–3185.

[26] De Luca G, Rivieccio G, Corsaro S. Value-at-Risk dynamics: a copula-VAR approach[J]. The European Journal of Finance, 2020, 26(2–3): 223–237.

[27] Chen C W S, Than-Thi H, So M K P, et al. Quantile forecasting based on a bivariate hysteretic autoregressive model with GARCH errors and time-varying correlations[J]. Applied Stochastic Models in Business and Industry, 2019, 35(6): 1301–1321.

[28] Stolbov M, Shchepeleva M. Macrofinancial linkages in Europe: Evidence from quantile local projections[J]. International Journal of Finance & Economics, 2021, 26(4): 5557–5569.

[29] Yang L, Yang L, Cui X. Sovereign default network and currency risk premia[J]. Financial Innovation, 2023, 9(1): 83. doi: 10.1186/s40854-023-00485-3 37192902 PMC10156581

[30] Yang L. Oil price bubbles: The role of network centrality on idiosyncratic sovereign risk[J]. Resources Policy, 2023, 82: 103493.

[31] Yang L, Cui X, Yang L, et al. Risk spillover from international financial markets and China’s macro-economy: A MIDAS-CoVaR-QR model[J]. International Review of Economics & Finance, 2023, 84: 55–69.

[32] Yang L. Connectedness of economic policy uncertainty and oil price shocks in a time domain perspective[J]. Energy Economics, 2019, 80: 219–233.

[33] Trung NB. The spillover effects of US economic policy uncertainty on the global economy: A global VAR approach[J]. The North American Journal of Economics and Finance, 2019, 48: 90–110.

[34] Zhang P, Gao J, Zhang Y, et al. Dynamic spillover effects between the US stock volatility and China’s stock market crash risk: a TVP-VAR approach[J]. Mathematical Problems in Engineering, 2021, 2021: 1–12.

[35] Chen J, Huang Y, Ren X, et al. Time-varying spillovers between trade policy uncertainty and precious metal markets: Evidence from China-US trade conflict[J]. Resources Policy, 2022, 76: 102577.

[36] Qiao X, Zhu H, Zhang Z, et al. Time-frequency transmission mechanism of EPU, investor sentiment and financial assets: A multiscale TVP-VAR connectedness analysis[J]. The North American Journal of Economics and Finance, 2022, 63: 101843.

[37] Cao G, Xie W. Asymmetric dynamic spillover effect between cryptocurrency and China’s financial market: Evidence from TVP-VAR based connectedness approach[J]. Finance Research Letters, 2022, 49: 103070.

[38] Liu L. US Economic uncertainty shocks and china’s economic activities: A time-varying perspective[J]. Sage Open, 2021, 11(3): 21582440211032672.

[39] Haddadan S, Zhuang Y, Cousins C, et al. Fast Doubly-Adaptive MCMC to Estimate the Gibbs Partition Function with Weak Mixing Time Bounds[J]. Advances in Neural Information Processing Systems, 2021, 34: 25760–25772.

[40] Oyeleke O J, Oyelami L O, Ogundipe A A. Investigating the monetary and fiscal policy regimes dominance for inflation determination in Nigeria: a Bayesian TVP-VAR analysis[J]. International Journal of Computational Economics and Econometrics, 2022, 12(3): 223–240.

[41] Almeida DB, Borges C L T, Oliveira GC, et al. Multi-area reliability assessment based on importance sampling, MCMC and stratification to incorporate variable renewable sources[J]. Electric Power Systems Research, 2021, 193: 107001.

[42] Fofack AD, Aker A, Rjoub H. Assessing the post-quantitative easing surge in financial flows to developing and emerging market economies[J]. Journal of Applied Economics, 2020, 23(1): 89–105.

[43] Hu Z, Kutan AM, Sun PW. Is US economic policy uncertainty priced in China’s A-shares market? Evidence from market, industry, and individual stocks[J]. International Review of Financial Analysis, 2018, 57: 207–220.

